# Supplementary material for: Alternative splicing modulation by G-quadruplexes
Source: Nat Commun. 2022 May 3;13:2404. doi: 10.1038/s41467-022-30071-7 (PMC9065059; doi:10.1038/s41467-022-30071-7)
Supplement: Supplementary file 1 — Supplementary Information [file 41467_2022_30071_MOESM1_ESM.pdf]

Supplementary Material

Supplementary Figure 1

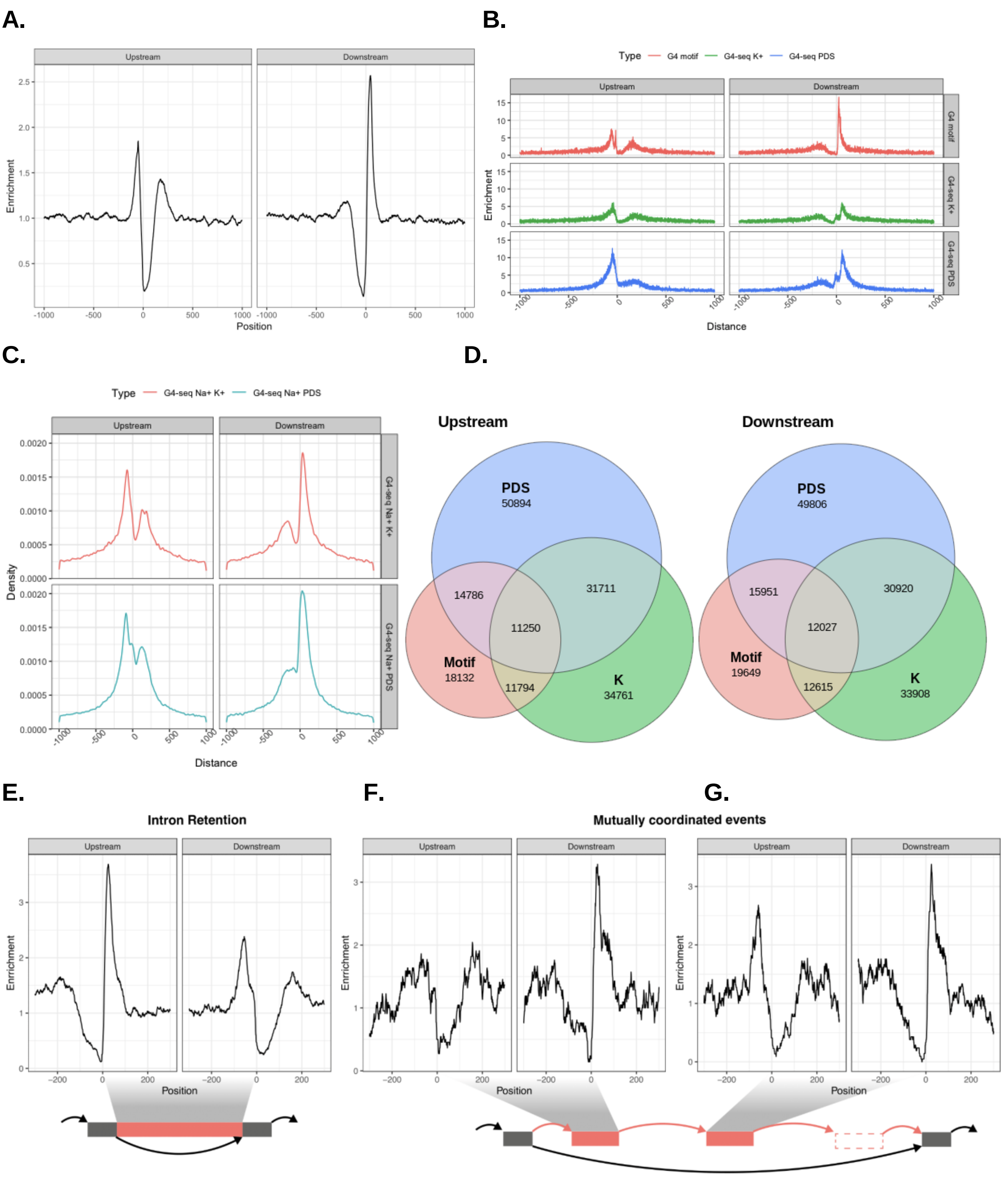

Supplementary Figure 1: Distribution of DNA G4s in the vicinity of splice sites. A. Distribution of G4 motifs at upstream and downstream splice sites in mouse. **B.** Enrichment between nearest G4 motif / G4-seq derived DNA G4 peak and a splice site separately for upstream and downstream splice sites using G4-seq datasets from Marsico et al.[51]. **C.** Distribution of G4-seq derived DNA G4 peaks within 1kB of the upstream and downstream splice sites after treatment with Na^+-PDS and Na^+-K^+. **D.** Venn diagrams for the number of G4 motifs found in a 100 nt window at the upstream and downstream splice sites that overlap G4-

seq derived DNA G4 peaks after treatment with Na^{+}-K^{+} and Na^{+}-PDS. G4-seq data with Na^{+}-PDS and Na^{+}-K^{+} treatments were taken from Chambers et al.[50]. **E.** Distribution of G4 motifs relative to intron retention sites. **F-G.** Distribution of G4 motifs relative to mutually coordinated events.

Supplementary Figure 2

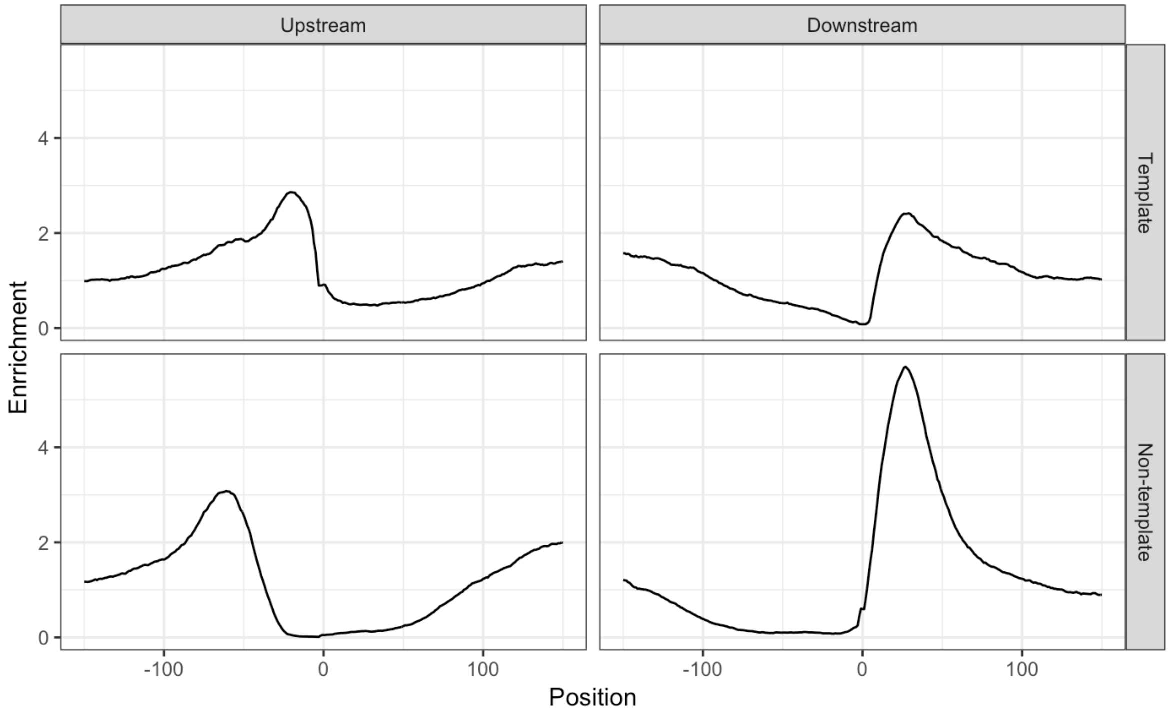

Supplementary Figure 2: The distribution of DNA G4s at splice junctions differs between the template and non-template strands. Distribution of G4 motifs at template and non-template strands at upstream (5’ss) and downstream (3’ss).

Supplementary Figure 3

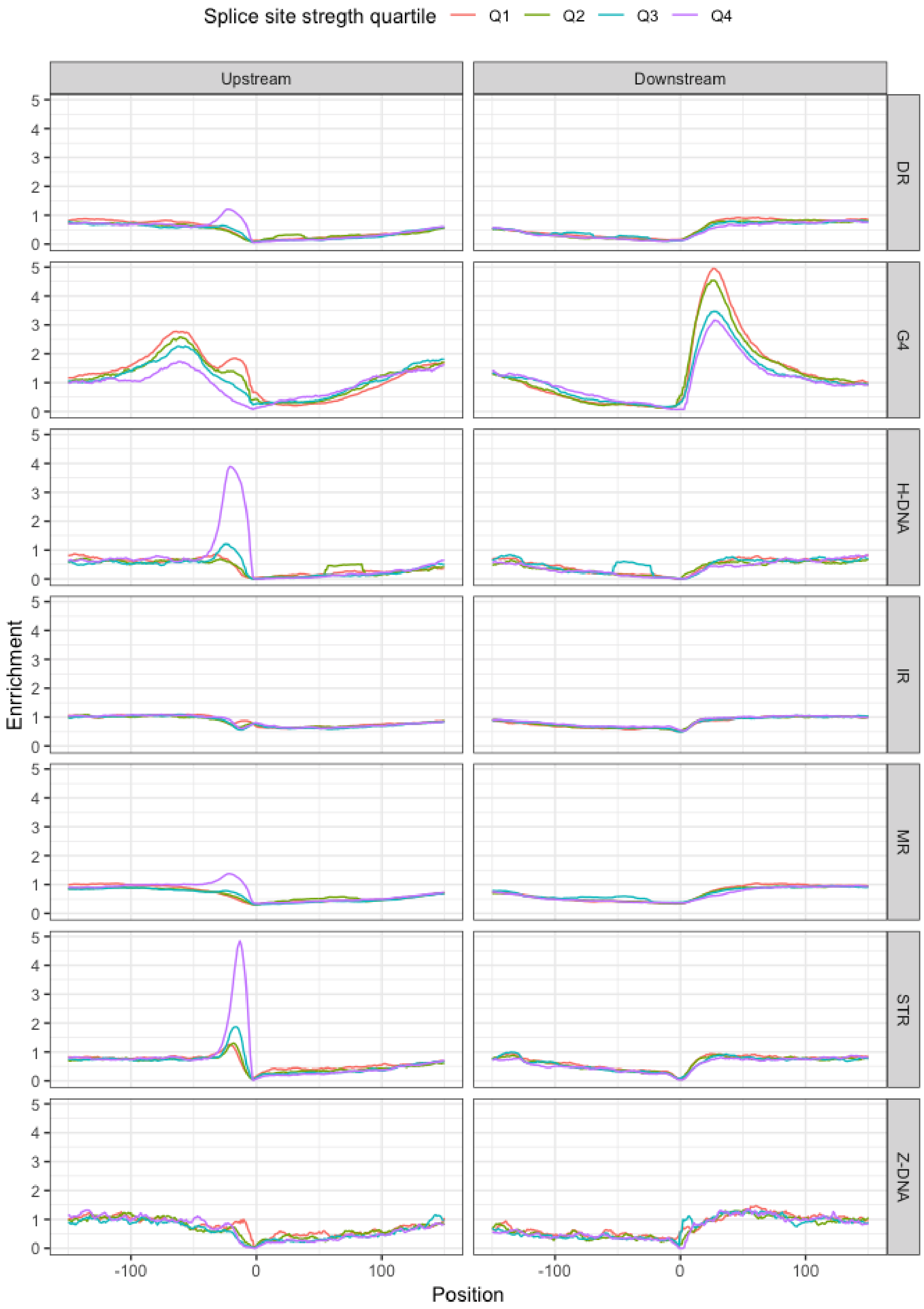

Supplementary Figure 3: Splice site strength and distribution of non-B DNA motifs at splicing sites. Dependence upon the splicing strength for seven non-B DNA motifs and their distribution at splicing sites relative to the upstream (3′ss) and the downstream (5′ss).

Supplementary Figure 4

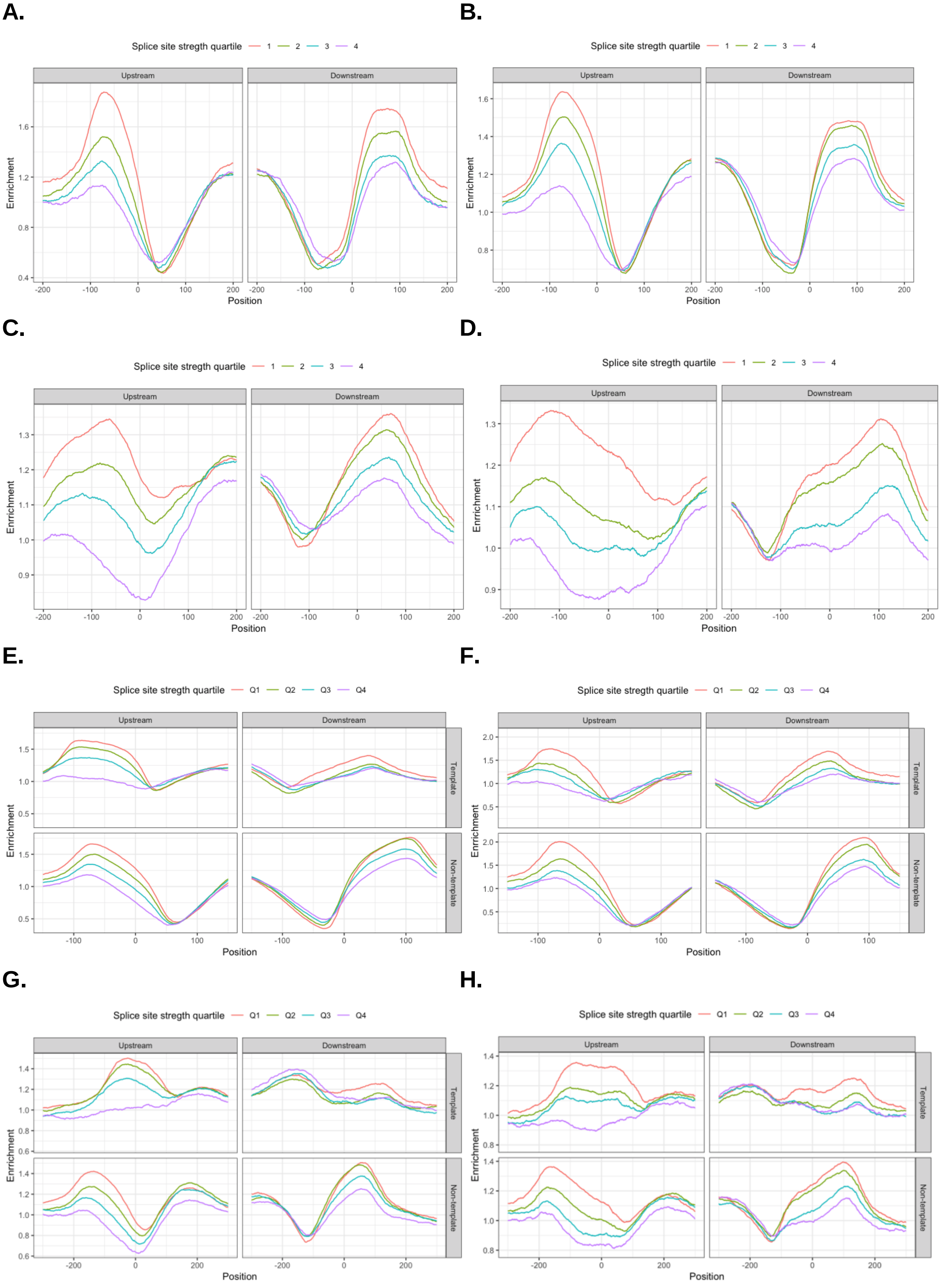

Supplementary Figure 4: Splice site strength and distribution of G4 motifs at splicing sites. G4s display a stronger enrichment at weaker splice sites. A. Distribution of DNA G4 peaks derived from G4-seq with K^+ treatment from Marsico et al.[51] at splicing sites at upstream (3′ss) and downstream (5′ss) and the association with splicing strength. **B.** Distribution of DNA G4 peaks derived from G4-seq with PDS treatment from Marsico et al.[51]

at upstream (3′ss) and downstream (5′ss) and the association with splicing strength. **C.** Distribution of DNA G4 peaks derived from G4-seq with Na^+-K^+ treatment from Chambers et al.[50] at upstream (3′ss) and downstream (5′ss) and the association with splicing strength. **D.** Distribution of DNA G4 peaks derived from G4-seq with Na^+-K^+ treatment from Chambers et al.[50] at upstream (3′ss) and downstream (5′ss) and the association with splicing strength. **E.** Distribution of DNA G4 peaks derived from G4-seq in presence of PDS at template and non-template strands and the association with splicing strength. **F.** Distribution of DNA G4 peaks derived from G4-seq in presence of K^+ at template and non-template strands and the association with splicing strength. **G.** Distribution of DNA G4 peaks derived from G4-seq in presence of Na^+-PDS at template and non-template strands and the association with splicing strength. **H.** Distribution of DNA G4 peaks derived from G4-seq in presence of Na^+-K^+ at template and non-template strands and the association with splicing strength. A-B and E-F panels used recent G4-seq data from Marsico et al.[51] with higher resolution than C-D and G-H panels which used older G4-seq data from Chambers et al.[50]. The median peak length for G4-seq peaks from Chambers et al.[50] was 255 nt and 195 nt in Na^+-K^+ and Na^+-PDS conditions, respectively. The median peak length for G4-seq peaks from Marsico et al.[51] was 135 nt and 120 nt in PDS and K^+ conditions, respectively.

Supplementary Figure 5

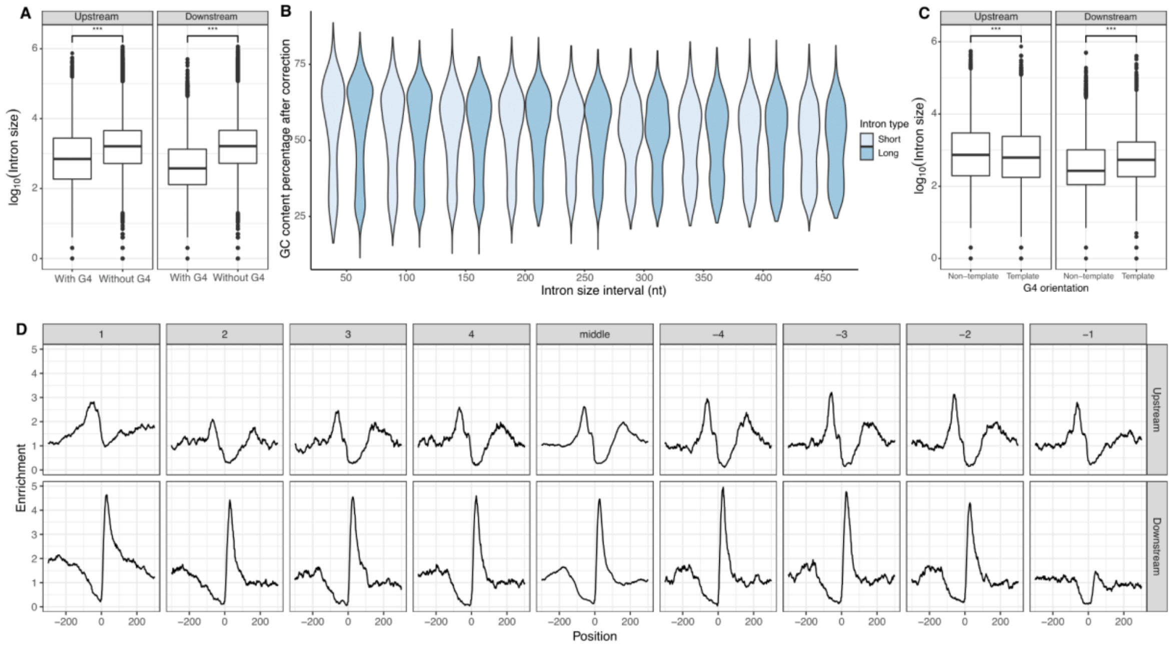

Supplementary Figure 5: A. Intron size of the upstream and downstream introns was calculated for groups with or without a G4 within 100 bps of the splice site (two-sided Mann-Whitney U p-value $<10^{-15}$ for both upstream and downstream introns). **B.** GC content distribution across selected groups of short and long introns. Intron size interval refers to the size of small introns. Long introns were defined as introns > 500 bp. **C.** Intron size for introns with template and non-template G4s within 100bp in the intronic region, for upstream and downstream splice sites (two-sided Mann-Whitney U p-value $<10^{-15}$ for both upstream and downstream introns). Adjusted p-values displayed as * for p-value<0.05, ** for p-value<0.01 and *** for p-value<0.001. **D**. G4 motif enrichment relative to the splice site across exons in the gene body at the 3′ss (upstream) and at the 5′ss (downstream). G4 motifs are enriched at both upstream (3′ss) and downstream (5′ss) across splice sites throughout the gene body. Exons were separated into first to fourth exons, middle exons, last four exons and the distribution of G4s were studied individually at each of them. For boxplots shown in A and C, the middle line corresponds to the median. The lower and upper boundaries of the box indicate the 25th and 75th percentiles respectively. The lower and upper boundaries of the whiskers indicate the 5th and 95th percentile respectively, and introns with sizes out of this range are shown as dots.

Supplementary Figure 6

A.

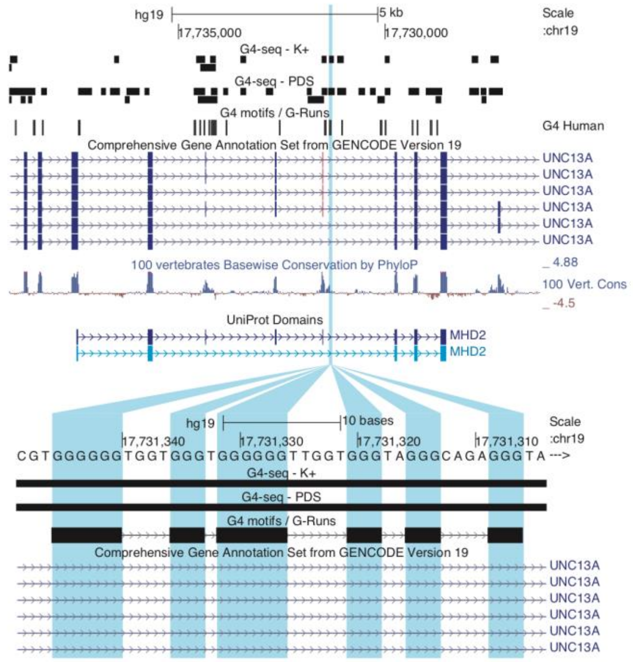

B.

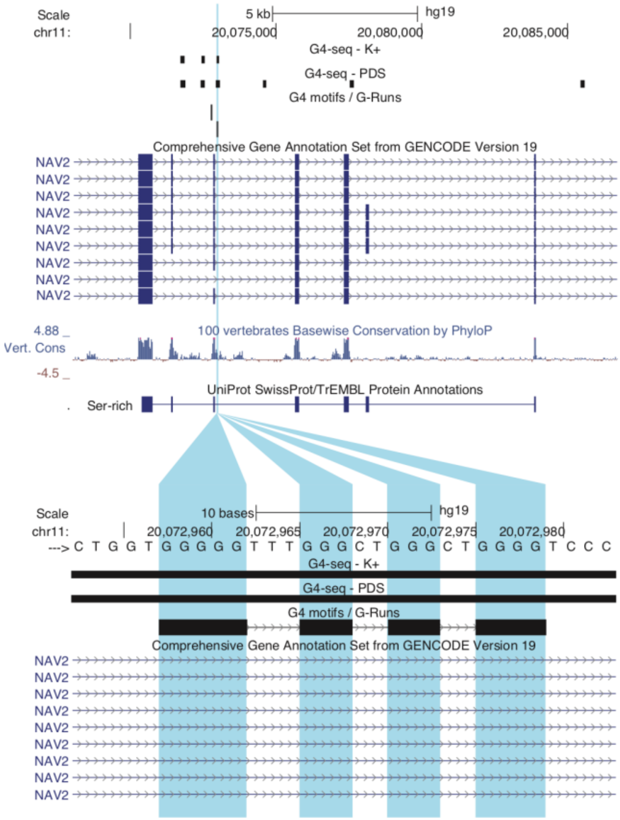

Supplementary Figure 6: Illustration of two loci with exons flanked by G4s, displaying alternative splicing changes following KCl treatment. A. Schematic of *UNC13A* exon 38 and the G4s around its splice junction. **B.** Schematic of *NAV2* exon 16 and the G4s around its splice junction.

Supplementary Figure 7

A.

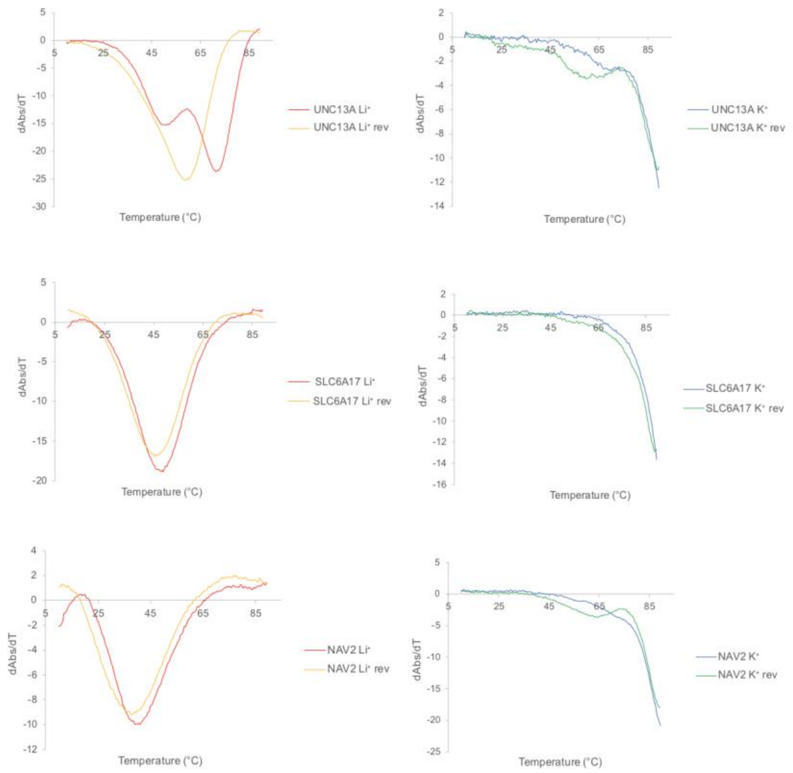

B.

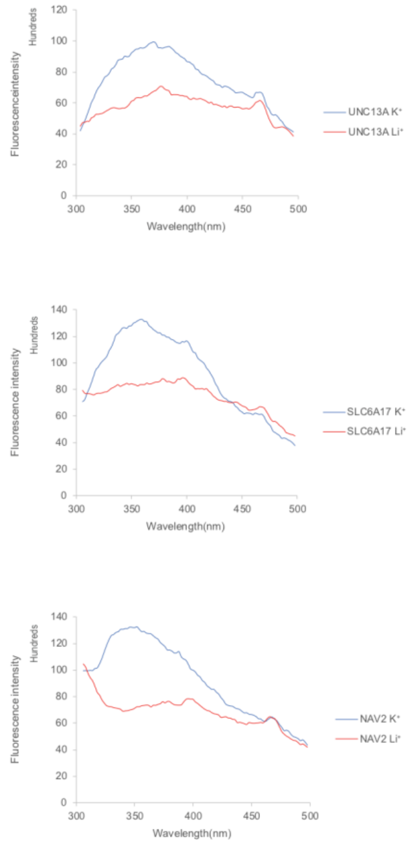

C.

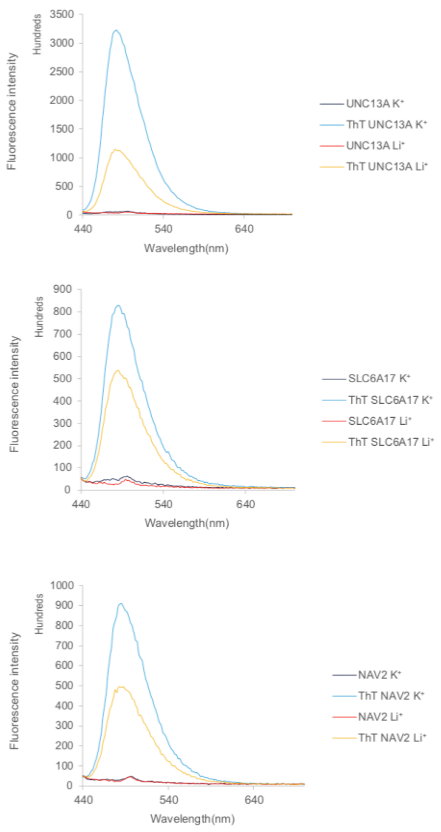

Supplementary Figure 7: G-quadruplex structure formation identification through fluorescence emission in presence of G4 stabilising (K^+) and non-stabilising (Li^+) cations. A. UV melting profiles of the three RNA G4 candidates in presence of Li^+ and K^+. Both the forward and reverse melt were shown here **B.** Intrinsic fluorescence of the three candidate RNA oligonucleotides under Li^+ or K^+ conditions. The intrinsic fluorescence of RNA G4s was increased when replacing Li^+ with K^+, highlighting the formation of RNA G4s. **C.** ThT ligand enhanced fluorescence of the three candidate RNA oligonucleotides under Li^+ or K^+ condition. In the absence of ThT ligand, no fluorescence was observed at ~480nm. Upon ThT addition, weak fluorescence was observed under Li^+, which was substantially enhanced when substituted with K^+, supporting the formation of RNA G4s which allow recognition of ThT and enhance its fluorescence.

\

Supplementary Figure 8

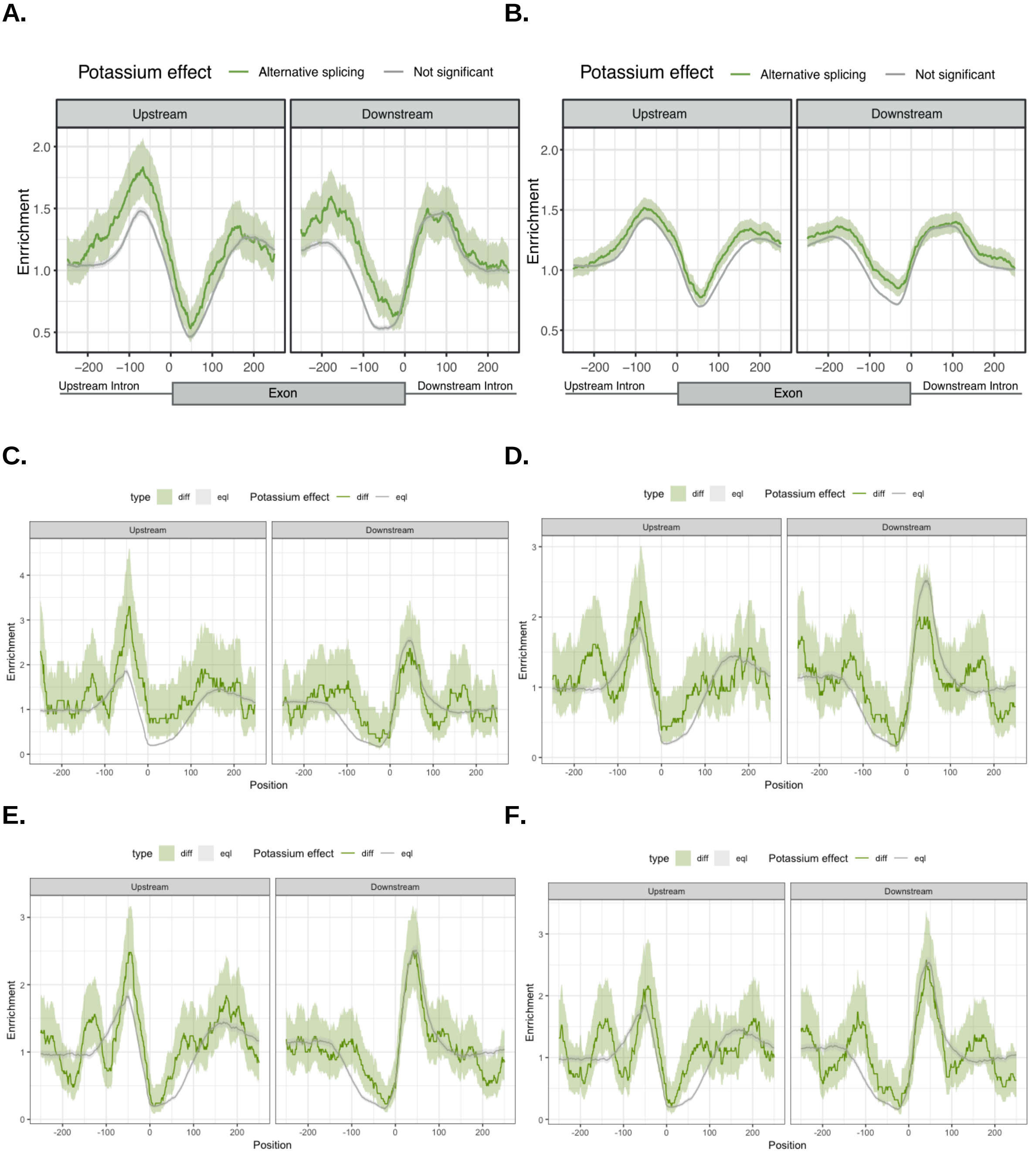

Supplementary Figure 8: Neuronal stimulation with KCl results in G4-mediated alternative splicing. A. Enrichment plots at 3′ / 5′ splice site vicinity after potassium stimulation of human neurons using G4-seq derived DNA G4 maps with PDS treatment. **B.** Enrichment plots at 3′ / 5′ splice site vicinity after potassium stimulation of human neurons using G4-seq derived DNA G4 maps with K^+ treatment. Splice sites with DNA G4 peaks within 100 nt were more likely to be differentially spliced following KCl treatment. **C.** Distribution of consensus G4 motifs at splicing sites after potassium depolarisation of CD1 mouse ESC-derived neurons. **D.** Distribution of consensus G4 motifs at splicing sites after potassium depolarisation of Tc1 mouse neurons at DIV10. **E.** Distribution of consensus G4 motifs at splicing sites after potassium depolarisation of CD1 mouse neurons at DIV4. **F.** Distribution of consensus G4 motifs at splicing sites after potassium depolarisation of CD1 mouse neurons at DIV10. The error bands represent 0.95 confidence intervals.

Supplementary Figure 9

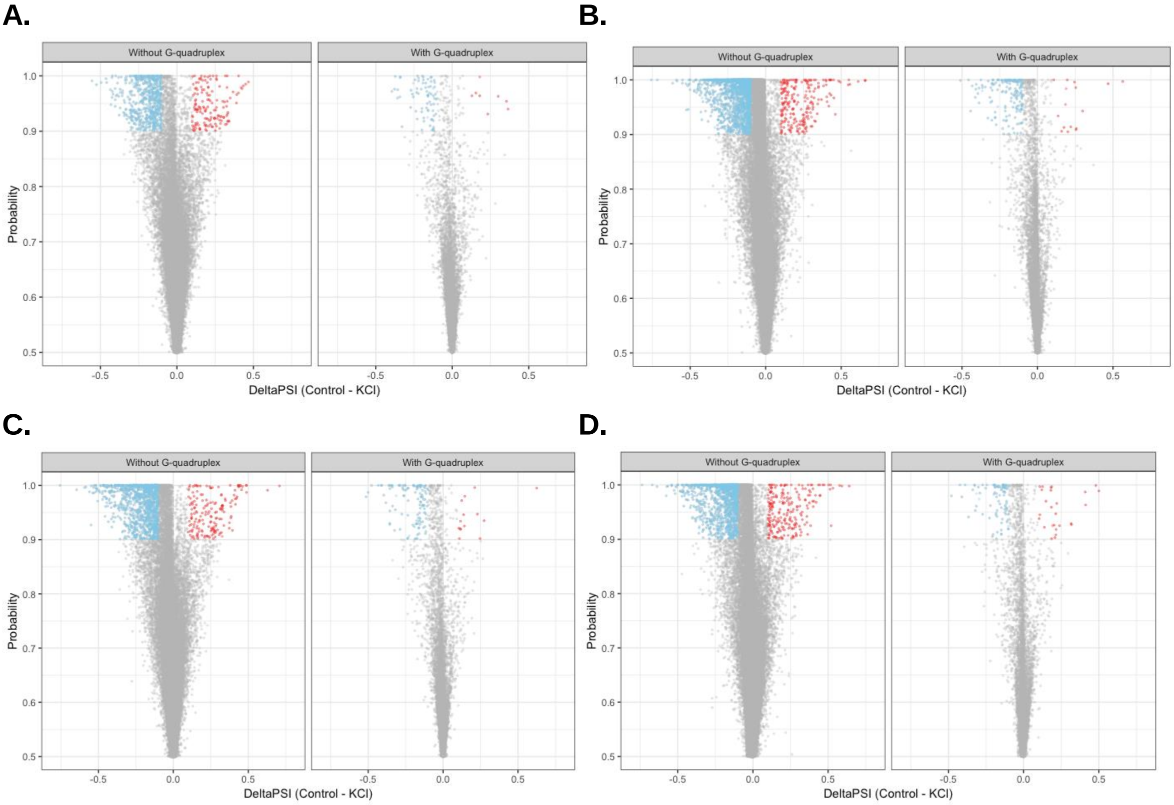

Supplementary Figure 9: Neuronal stimulation with KCl results in exon skipping in human and mouse neuronal cells. Volcano plots for **A.** CD1 mouse ESC-derived neurons, **B.** CD1 mouse primary cortical neurons at DIV4, **C.** CD1 mouse primary cortical neurons at DIV10, **D.** Tc1 mouse primary cortical neurons at DIV10. We found consistent exon skipping of canonical exons following KCl treatment in all cases (Chi-squared test with Bonferroni correction: p-value<0.001 in all cases).

Supplementary Figure 10

A.

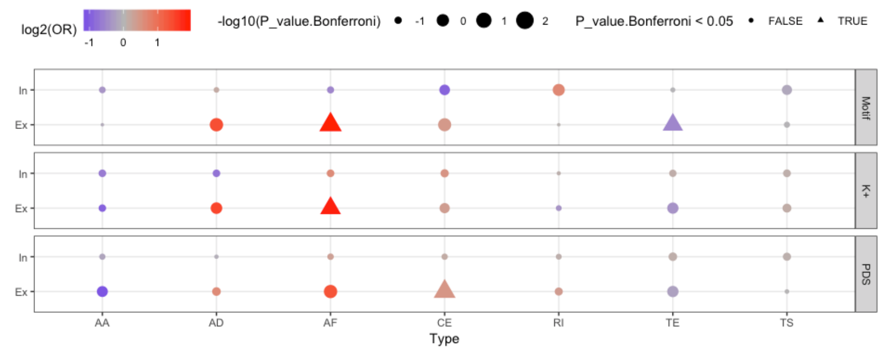

B.

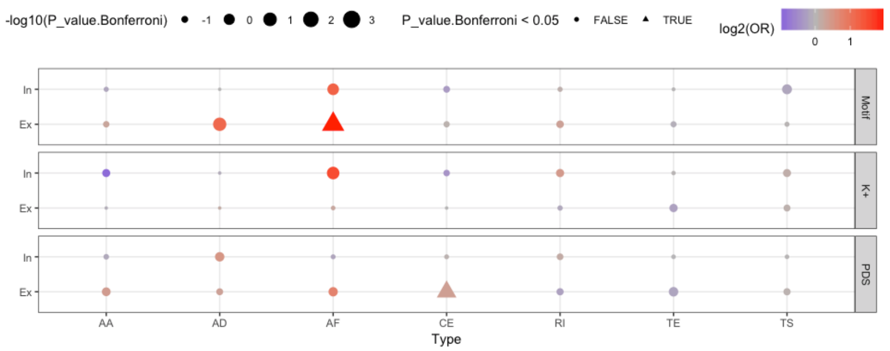

C.

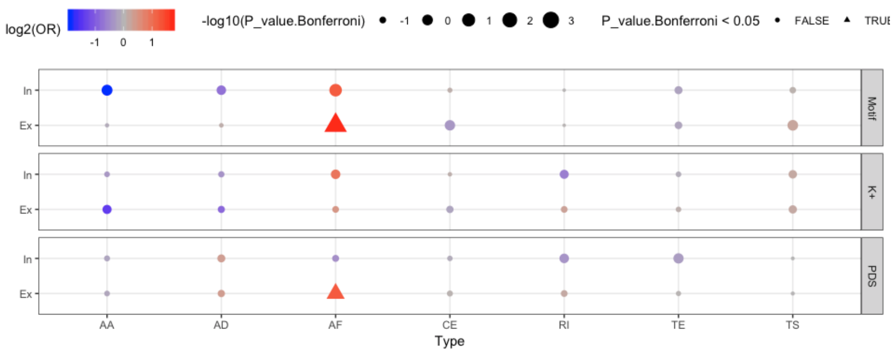

D.

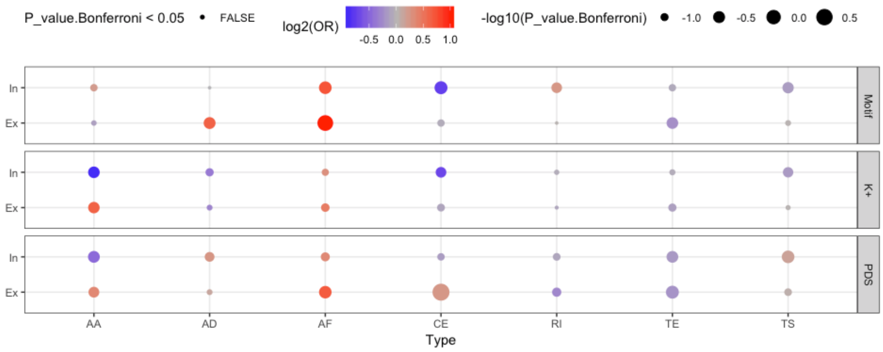

Supplementary Figure 10: Inclusion and exclusion patterns of genic regions in association to G4 presence or absence following KCl treatment. All p-values were obtained with a chi-squared test and were Bonferroni corrected. **A.** ESC-derived neurons from CD1 mice. **B.** Primary cortical neurons from DIV10 CD1 mice. **C.** Primary cortical neurons from DIV10 Tc1 mice. **D.** Primary cortical neurons from DIV4 CD1 mice. Types correspond to:

AA - Alternative Acceptor Splice Site, AD - Alternative Donor Splice Site, AF - Alternative First Exon, CE - Core Exon, TE - Tandem Alternative Polyadenylation Site, TS - Tandem Transcription Start Site.

Supplementary Figure 11

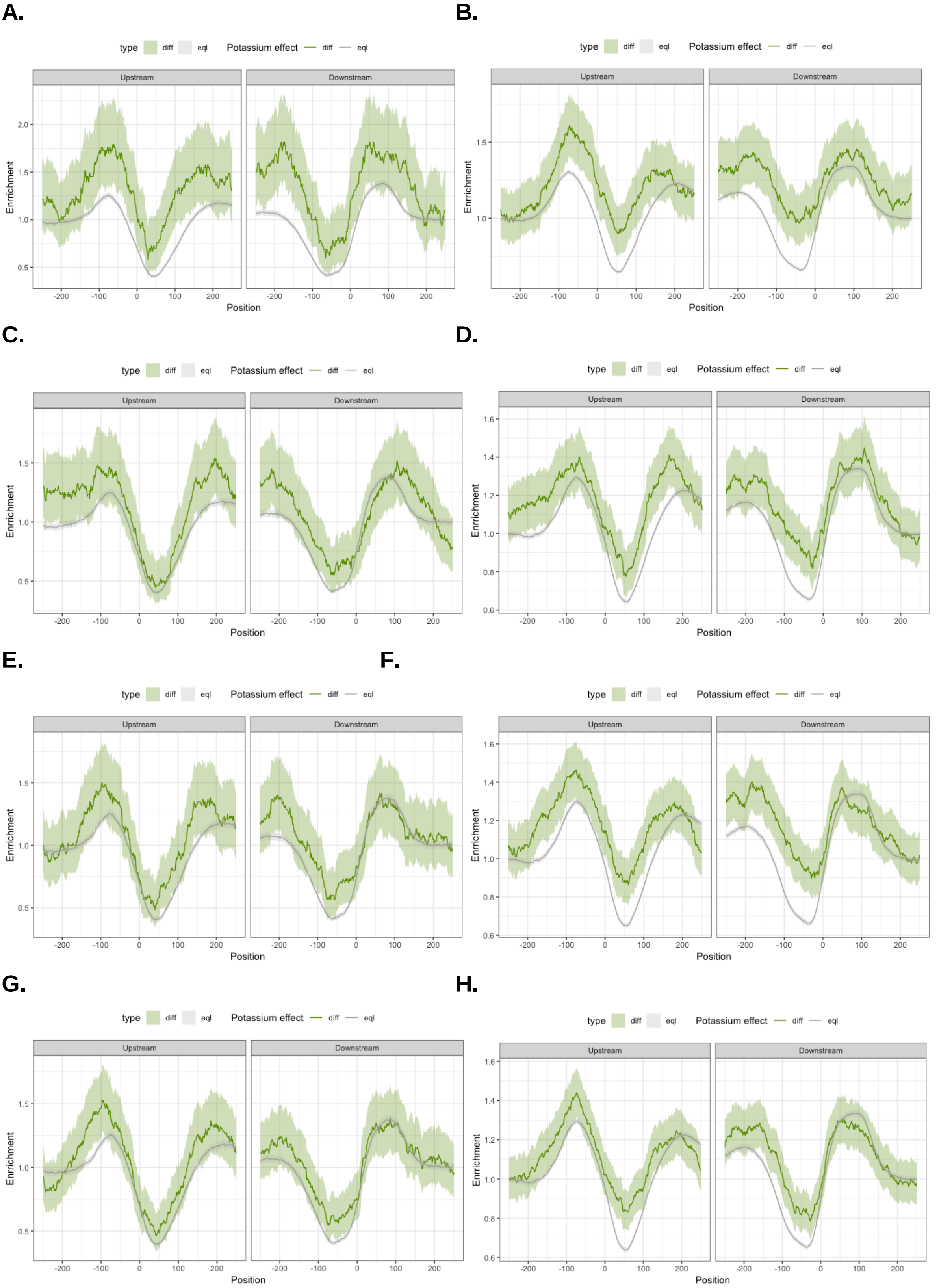

Supplementary Figure 11: Neuronal stimulation with KCl results in G4-mediated alternative splicing using G4-seq derived DNA G4 peaks. A. Distribution of G4-seq derived DNA G4 peaks with K^+ treatment at splicing sites after potassium depolarisation of CD1 mouse ESC-derived neurons. **B.** Distribution of G4-seq derived DNA G4 peaks with PDS treatment

at splicing sites after potassium depolarisation of CD1 mouse ESC-derived neurons. **C.** Distribution of G4-seq derived DNA G4 peaks with K^+ treatment at splicing sites after potassium depolarisation of Tc1 mouse neurons at DIV10. **D.** Distribution of G4-seq derived DNA G4 peaks with PDS treatment at splicing sites after potassium depolarisation of Tc1 mouse neurons at DIV10. **E.** Distribution of G4-seq derived DNA G4 peaks with K^+ treatment at splicing sites after potassium depolarisation of CD11 mouse neurons at DIV10. **F.** Distribution of G4-seq derived DNA G4 peaks with PDS treatment at splicing sites after potassium depolarisation of CD1 mouse neurons at DIV10. **G.** Distribution of G4-seq derived DNA G4 peaks with K^+ treatment at splicing sites after potassium depolarisation of CD1 mouse neurons at DIV4. **H.** Distribution of G4-seq derived DNA G4 peaks with PDS treatment at splicing sites after potassium depolarisation of CD1 mouse neurons at DIV4. The error bands represent 0.95 confidence intervals.

Supplementary Figure 12

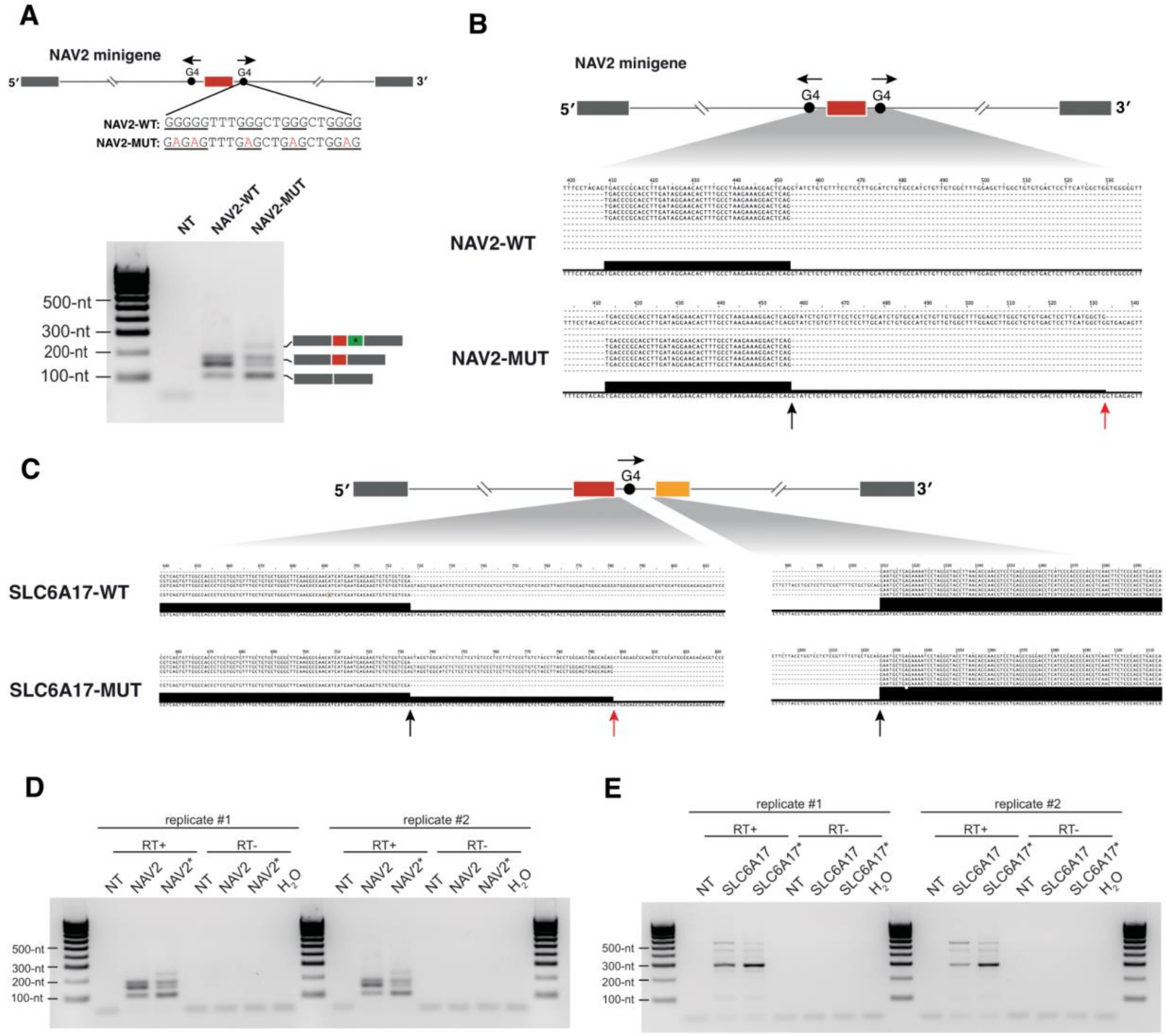

Supplementary Figure 12: Minigene experiments with wild-type and mutant G4 sequences flanking the splice site of the target exon. A. The schematic on top shows the design where a NAV2 exon that is flanked by two G4s was inserted into a minigene, arrows intricate the strand in which G4s were present. Downstream nontemplate G4 was mutated along its G-runs to prevent G4 formation. PCR followed by electrophoresis shows that NAV2 exon (red) is predominantly excluded after the introduced mutations. G4 mutations also activate a cryptic splice site that is not observed in wild-type sequences (green) and it was confirmed by Sanger sequencing. Double bands were observed to match with the expected weight of NAV2 including isoform, but no additional cryptic sites were detected by Sanger sequencing. **B-C.** Most expected alternative splicing products were confirmed by Sanger sequencing. Analysis of the sequencing results also corroborates the activation of cryptic splice sites upon the introduced mutations at target G4 sequences. Black and red indicate wild-type and cryptic splice sites respectively. **D-E.** Consistent band migration patterns were observed across experiment replicates that were performed after independent cell transfections (Replicate #1 and #2). PCR replicates also show consistent band migration patterns (top row and bottom row), showing a reproducible effect of mutations over downstream non-template G4s over alternative splicing.

Supplementary Figure 13

A.

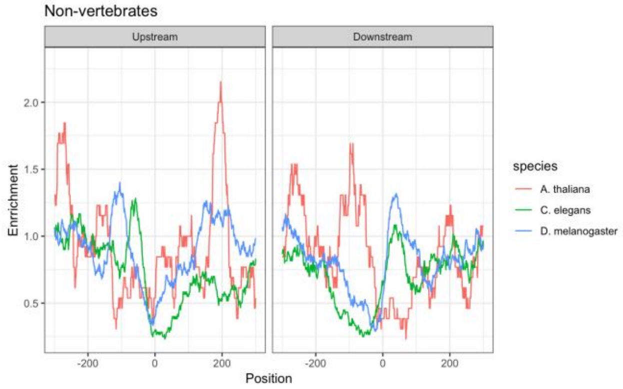

B.

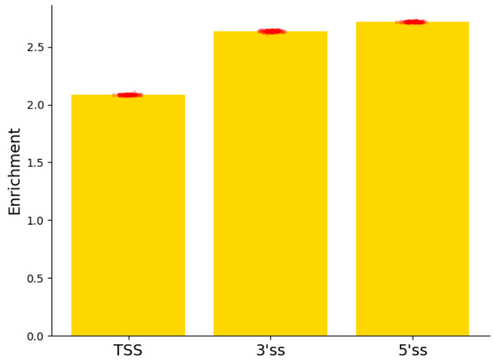

Supplementary Figure 13: Distribution of G4s at splicing sites across multiple species. A. Enrichment of consensus G4 motifs at splicing sites across different non-vertebrate species. Enrichment indicates the frequency of G4s at a position over the median frequency across the window. **B.** Enrichment (Observed / Expected) of consensus G4 motifs at 3′ss, 5′ss and TSS for humans. Observed frequency was calculated at 100 nt windows on each side of the upstream (3′ss), downstream (5′ss) splice sites and TSS sites and expected frequency was calculated across 100-fold simulations of all 100 nt windows controlling for dinucleotide content with the error bars representing the standard deviation. Red dots represent the enrichment scores from the simulations.

Supplementary Figure 14

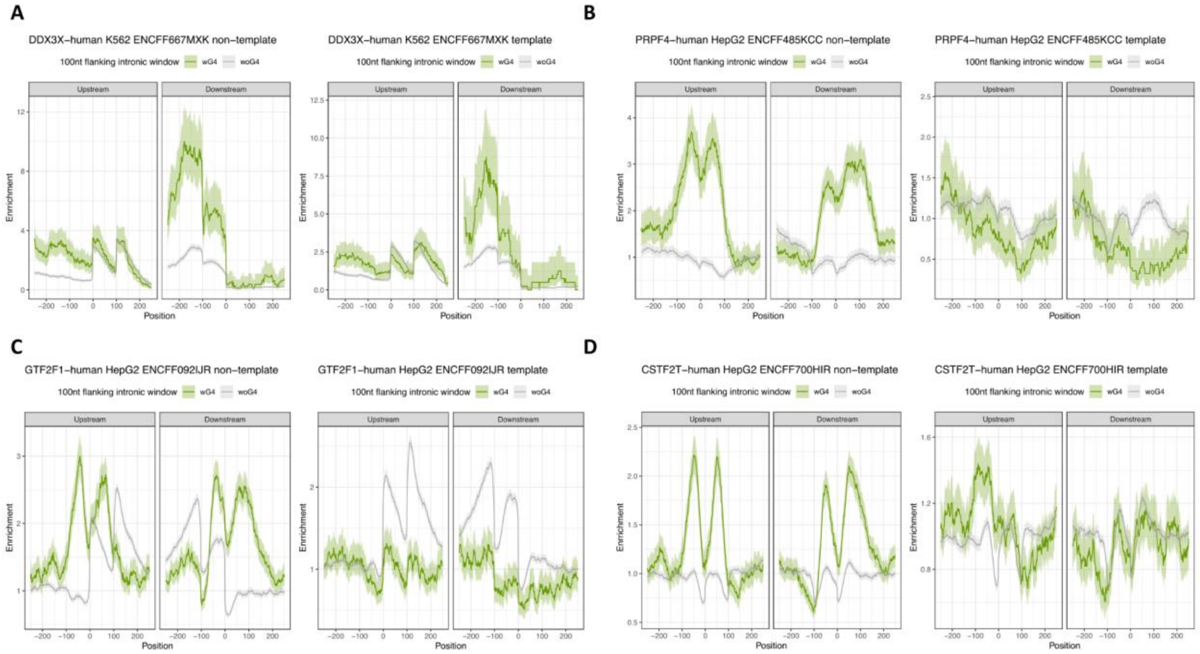

Supplementary Figure 14: Enrichment of a panel of RBP binding sites across splice sites flanked or not flanked by G4s. Displayed factors are **A.** DDX3X, B. PRPF4, **C**. GTF2F1, **D.** CSTF2T, each of which belongs to a different eCLIP cluster. The error bands represent 95% confidence intervals based on a binomial model.
