# Supplementary material for: Alternative splicing modulation by G-quadruplexes
Source: Nat Commun. 2022 May 3;13:2404. doi: 10.1038/s41467-022-30071-7 (PMC9065059; doi:10.1038/s41467-022-30071-7)
Supplement: Supplementary file 2 — Reporting Summary [file 41467_2022_30071_MOESM2_ESM.pdf]

nature portfolio

Corresponding author(s): Martin Hemberg

Last updated by author(s): Mar 28, 2022

Reporting Summary

Nature Portfolio wishes to improve the reproducibility of the work that we publish. This form provides structure for consistency and transparency in reporting. For further information on Nature Portfolio policies, see our Editorial Policies and the Editorial Policy Checklist.

Statistics

For all statistical analyses, confirm that the following items are present in the figure legend, table legend, main text, or Methods section.

n/a	Confirmed	
☐	☒	The exact sample size (*n*) for each experimental group/condition, given as a discrete number and unit of measurement
☐	☒	A statement on whether measurements were taken from distinct samples or whether the same sample was measured repeatedly
☐	☒	The statistical test(s) used AND whether they are one- or two-sided<br>*Only common tests should be described solely by name; describe more complex techniques in the Methods section.*
☒	☐	A description of all covariates tested
☐	☒	A description of any assumptions or corrections, such as tests of normality and adjustment for multiple comparisons
☐	☒	A full description of the statistical parameters including central tendency (e.g. means) or other basic estimates (e.g. regression coefficient) AND variation (e.g. standard deviation) or associated estimates of uncertainty (e.g. confidence intervals)
☐	☒	For null hypothesis testing, the test statistic (e.g. *F*, *t*, *r*) with confidence intervals, effect sizes, degrees of freedom and *P* value noted<br>*Give P values as exact values whenever suitable.*
☒	☐	For Bayesian analysis, information on the choice of priors and Markov chain Monte Carlo settings
☐	☒	For hierarchical and complex designs, identification of the appropriate level for tests and full reporting of outcomes
☐	☒	Estimates of effect sizes (e.g. Cohen's *d*, Pearson's *r*), indicating how they were calculated

Our web collection on statistics for biologists contains articles on many of the points above.

Software and code

Policy information about availability of computer code

Data collection	Public data used in this study were obtained from the ENCODE consortium, the GTEx consortium, Cer et al. 2013 (v2.0), BioProject Accession PRJEB19451 with ENA link ERP021488 and data from accession codes GSE110582 and GSE63874. Data were also collected from Jasco J-1500 CD spectrophotometer and HORBIA FluoroMax-4.
Data analysis	Data were preprocessed with custom code that can be found at https://github.com/hemberg-lab/Georgakopoulos_Soares_and_Parada_2022. Data processing and analysis was also performed with open source available software including Whippet (https://github.com/timbitz/Whippet.jl), "ggsashimi" package, "Hmisc" package HISAT2 (v2.1.0), DEseq2 (v1.18.1) a Snakemake pipeline (Köster and Rahmann 2012) that was adapted from a publicly available repository (Köster et al. 2021), FeatureCounts (Liao, Smyth, and Shi 2014).

For manuscripts utilizing custom algorithms or software that are central to the research but not yet described in published literature, software must be made available to editors and reviewers. We strongly encourage code deposition in a community repository (e.g. GitHub). See the Nature Portfolio guidelines for submitting code & software for further information.

Data

Policy information about availability of data

All manuscripts must include a data availability statement. This statement should provide the following information, where applicable:

- Accession codes, unique identifiers, or web links for publicly available datasets
- A description of any restrictions on data availability
- For clinical datasets or third party data, please ensure that the statement adheres to our policy

The data of this manuscript have been uploaded to Zenodo with DOI: 10.5281/zenodo.6324564

All the associated code used for the generation of figures and presentation of data throughout the manuscript is deposited in GitHub at the following link: https://github.com/hemberg-lab/Georgakopoulos_Soares_and_Parada_2022

Field-specific reporting

Please select the one below that is the best fit for your research. If you are not sure, read the appropriate sections before making your selection.

☒ Life sciences ☐ Behavioural & social sciences ☐ Ecological, evolutionary & environmental sciences

For a reference copy of the document with all sections, see nature.com/documents/nr-reporting-summary-flat.pdf

Life sciences study design

All studies must disclose on these points even when the disclosure is negative.

Sample size	Due to time and budget restrictions, two candidates were selected for the construction of minigene experiments. Two loci were selected for the construction of minigene validation experiments. Two independent biological replicates were performed, which is a standard consideration for this type of molecular biology experiments.
Data exclusions	Not applicable.
Replication	For the minigene experiment two independent transfections were performed on two different batches of the selected cell line. Subsequently, two independent RT-PCR experiments were performed after the RNA extraction from the two independent biological replicates.
Randomization	Not applicable.
Blinding	Not applicable.

Reporting for specific materials, systems and methods

We require information from authors about some types of materials, experimental systems and methods used in many studies. Here, indicate whether each material, system or method listed is relevant to your study. If you are not sure if a list item applies to your research, read the appropriate section before selecting a response.

Materials & experimental systems

n/a	Involved in the study	
☒	☐	Antibodies
☐	☒	Eukaryotic cell lines
☒	☐	Palaeontology and archaeology
☒	☐	Animals and other organisms
☒	☐	Human research participants
☒	☐	Clinical data
☒	☐	Dual use research of concern

Methods

n/a	Involved in the study	
☒	☐	ChIP-seq
☒	☐	Flow cytometry
☒	☐	MRI-based neuroimaging

Eukaryotic cell lines

Policy information about cell lines

Cell line source(s)	DU145 prostate cancer cell line.
Authentication	Not performed.
Mycoplasma contamination	The cell culture facilities regularly inspect for mycoplasma contamination, which has not been found.
Commonly misidentified lines (See ICLAC register)	None.
